# Supplementary material for: Development of an ectopic huLiver model for Plasmodium liver stage infection
Source: PLoS One. 2023 Mar 16;18(3):e0279144. doi: 10.1371/journal.pone.0279144 (PMC10019673; doi:10.1371/journal.pone.0279144)
Supplement: S1 File — Development of an Ectopic huLiver Model for Plasmodium Liver Stage Infection. (DOCX) [file pone.0279144.s001.docx]

**Supporting Methods**

**Development of an Ectopic huLiver Model for *Plasmodium* Liver Stage Infection**

Gabriela Samayoa-Reyes^¶1^, Siobhan M. Flaherty^¶1^, Kristina S. Wickham^2^, Sara Viera-Morilla^3^, Pamela M. Strauch^1^, Alison Roth^2^, Laura Padrón^3^, Conner M. Jackson^1^, Patricia Meireles^3^, David Calvo^3^, Wanlapa Roobsoong^4^, Niwat Kangwanrangsan^5^, Jetsumon Sattabongkot^4^, Gregory Reichard^2^, Maria José Lafuente-Monasterio^3^, Rosemary Rochford^1*^

^1^Department of Immunology and Microbiology, University of Colorado School of Medicine, Aurora, Colorado, USA.

^2^Department of Drug Discovery, Experimental Therapeutics Branch, Walter Reed Army Institute of Research, Silver Spring, Maryland, USA.

^3^Diseases of the Developing World, Infectious Diseases-Centre for Excellence in Drug Discovery (ID CEDD), GlaxoSmithKline, Tres Cantos, Madrid, Spain.

^4^Mahidol Vivax Research Unit, Faculty of Tropical Medicine, Mahidol University, Bangkok, Thailand.

^5^Pathobiology Department, Faculty of Science, Mahidol University, Bangkok, Thailand.

^*^ Corresponding author

**Email:** [rosemary.rochford@cuanschutz.edu](mailto:rosemary.rochford@cuanschutz.edu) (RR)

^¶^GSR and SF are co-first authors. These authors contributed equally to this work.

**Supporting Methods**

*Liver stage infection determination using P. berghei 18S qRT-PCR*: Ectopic huLiver and FRG-huHep mice were euthanized 44 hours post-infection. Both liver and ectopic huLiver tissue were harvested for qRT-PCR quantification and homogenized with RNA extracted and cDNA generated as previously described in the text. cDNA was used for the amplification of *P. berghei* 18S rRNA and the housekeeping gene Glyceraldehyde-3-Phosphate Dehydrogenase (hu*GAPDH)*. *P. berghei* 18S rRNA was detected using the following primers rRNA F: 5- CTG GTT GAT CTT GCC AGT AG- 3 and rRNA R: 5- GGA TAA CTA CGG AAA AGC TGT A-3 and sample quality verified using the housekeeping gene *GAPDH.* Reactions were run in a ViiA 7 Real-Time PCR system (Applied Biosystems, Foster City, CA) using the reaction presets. Reverse transcriptase negative and non-template controls were used.

*HC-04 cell line modifications* The HC-04 EphA2^High^ CD81 ^High^ cell line was created using lentiviral vectors (LV) that expressed either EphA2 or CD81, the LVs were used to infect HC-04 cells, and high expressing cells were selected for through the use of cell sorting techniques. HC-04 (ATCC MRA-975) and HC-04 EphA2^High^ CD81 ^High^ cell lines were cultured in a 1:1 Dulbecco’s Modified Eagle’s Medium (DMEM): Ham’s F12 media (Invitrogen, Carlsbad, CA) supplemented with 10% (v/v) fetal bovine serum (FBS), 1% glutamine, 100 U/mL penicillin and 100 ug/mL streptomycin. Cultures were maintained at 37°C in a humidified atmosphere with 5% CO_2_. Cells were sub-cultured every 2-4 days until reaching approximately 70% confluency. Cell detachment was performed using 0.125% trypsin (Invitrogen, Carlsbad, CA).

*HC-04 modified cell line EphA2 and CD81 levels in vivo* Five ectopic huLiver mice were euthanized 24 days post cell engraftment. Ectopic huLivers were harvested for immunofluorescence and qRT-PCR analysis.

*Immunofluorescence staining of EPHA2:* Uninfected HC-04 and HC-04 modified ectopic huLiver tissue was processed and stained using methods described above with mouse IgG2a anti-EphA2 APC-conjugated antibody (R&D Systems, Minneapolis, MN) (1:100) and a polyclonal rabbit anti-laminin Dylight 488 antibody (Invitrogen, Carlsbad, CA) (1:100).

*qRT-PCR.* A 30 mg section of the ectopic huLiver was homogenized and RNA extraction was performed using the RNeasy Plus Mini Kit (Qiagen, Hilden, Germany), following the manufacturer’s protocol. The isolated RNA was quantified using a NanoDrop spectrophotometer (Thermo Fisher, Waltham, MA). RNA was reverse transcribed using the High-Capacity cDNA Reverse Transcription Kit (Applied Biosystems, Foster City, CA). The resulting cDNA was used for amplification using SYBR Green kit (Applied Biosystems, Foster City, CA). Expression of EphA2 was determined with the primers previously described [57]. Expression of CD81 was examined by qRT-PCR analysis using the following primers CD81 F: 5- GTC TTC AAT TTC GTC TTC TGG-3 and R: 5- GTA GGC ATC TAC ATC CTC ATC G-3. Human *GAPDH,* was used as housekeeping gene [58]. Samples were run in triplicate and each transcript was normalized to *GAPDH* (deltaCt). 2^–∆∆Ct^ formula was then used to calculate mRNA fold-change relative to untreated cells [59].

**References**

57. Chen J, Sathiyamoorthy K, Zhang X, Schaller S, Perez White BE, Jardetzky TS, Longnecker R. Ephrin receptor A2 is a functional entry receptor for Epstein–Barr virus. Nat Microbiol. 2018 Feb;3(2):172–80.

58. Painter MM, Morrison JH, Zoecklein LJ, Rinkoski TA, Watzlawik JO, Papke LM, Warrington AE, Bieber AJ, Matchett WE, Turkowski KL, Poeschla EM, Rodriguez M. Antiviral Protection via RdRP-Mediated Stable Activation of Innate Immunity. Ross SR, editor. PLoS Pathog. 2015 Dec 3;11(12):e1005311.

59. Schmittgen TD, Livak KJ. Analyzing real-time PCR data by the comparative CT method. Nat Protoc. 2008 Jun;3(6):1101–8.
