# Supplementary material for: Development of an ectopic huLiver model for Plasmodium liver stage infection
Source: PLoS One. 2023 Mar 16;18(3):e0279144. doi: 10.1371/journal.pone.0279144 (PMC10019673; doi:10.1371/journal.pone.0279144)
Supplement: S2 File — Development of an Ectopic huLiver Model for Plasmodium Liver Stage Infection. (DOCX) [file pone.0279144.s002.docx]

**Supporting Results**

**Development of an Ectopic huLiver Model for *Plasmodium* Liver Stage Infection**

Gabriela Samayoa-Reyes^¶1^, Siobhan M. Flaherty^¶1^, Kristina S. Wickham^2^, Sara Viera-Morilla^3^, Pamela M. Strauch^1^, Alison Roth^2^, Laura Padrón^3^, Conner M. Jackson^1^, Patricia Meireles^3^, David Calvo^3^, Wanlapa Roobsoong^4^, Niwat Kangwanrangsan^5^, Jetsumon Sattabongkot^4^, Gregory Reichard^2^, Maria José Lafuente-Monasterio^3^, Rosemary Rochford^1*^

^1^Department of Immunology and Microbiology, University of Colorado School of Medicine, Aurora, Colorado, USA.

^2^Department of Drug Discovery, Experimental Therapeutics Branch, Walter Reed Army Institute of Research, Silver Spring, Maryland, USA.

^3^Diseases of the Developing World, Infectious Diseases-Centre for Excellence in Drug Discovery (ID CEDD), GlaxoSmithKline, Tres Cantos, Madrid, Spain.

^4^Mahidol Vivax Research Unit, Faculty of Tropical Medicine, Mahidol University, Bangkok, Thailand.

^5^Pathobiology Department, Faculty of Science, Mahidol University, Bangkok, Thailand.

^*^ Corresponding author

**Email:** [rosemary.rochford@cuanschutz.edu](mailto:rosemary.rochford@cuanschutz.edu) (RR)

^¶^GSR and SF are co-first authors. These authors contributed equally to this work.

**Supporting Table 1. Ectopic huLiver mice infected with *P. beghei*-*luc*.**

| ***P. berghei*-Luc Sporozoites** | **qRT-PCR 44hpi**  **18S *P. berghei*-Luc** | |
| --- | --- | --- |
|  | Liver | Ectopic huLiver |
| **200,000** | Positive | Positive |
| **50,000** | Positive | Positive |
| **30,000** | Positive | Positive |

NSG ectopic huLiver mice (n =3) were intratumorally infected with varying amounts of *P. berghei-Luc* sporozoites, as indicated. 44 hrs post infection (hpi) mice were euthanized to determine infection by qRT-PCR. Here we show quantification of parasite burden by qRT-PCR of *P. berghei* 18S rRNA and reported as either positive or negative in both liver and ectopic huLiver tissue.

**Supporting Figure 1. Confirmation of NF54HT-Luc *P. falciparum* luciferase activity.** IVIS imagining of NSG mice that had been engrafted with huRBCs was done to confirm parasite luciferase activity.

**Supporting Figure 2. Characterization of the modified HC-04 cell line.** The HC-04 wild type (WT) cell line and HC-04 EphA2^High^ CD81 ^High^ modified cell line were compared to assess functionality and receptor expression. **(A)** Ectopic huLiver growth curve comparison. 5 X10^6^ cells of either HC-04 WT cell line or HC-04 EphA2^High^ CD81 ^High^ cell line were injected s.c. on the flank of NSG mice with ectopic huLiver growth monitored daily until day 22 post engraftment. **(B)** Ectopic huLiver immunofluorescence labeling. Liver sections were assayed by indirect immunofluorescence using the following antibodies: mouse EphA2 APC-conjugated antibody (yellow), polyclonal rabbit anti-laminin Dylight 488 antibody (green) and DNA was visualized using DAPI (blue). **(C)** EphA2 and CD81 expression levels. EphA2 and CD81 receptor expression levels measured by qRT-PCR, relative to the housekeeping gene *GAPDH.*
