# Supplementary figures and images for: Development of an ectopic huLiver model for Plasmodium liver stage infection
Source: PLoS One. 2023 Mar 16;18(3):e0279144. doi: 10.1371/journal.pone.0279144 (PMC10019673; doi:10.1371/journal.pone.0279144)

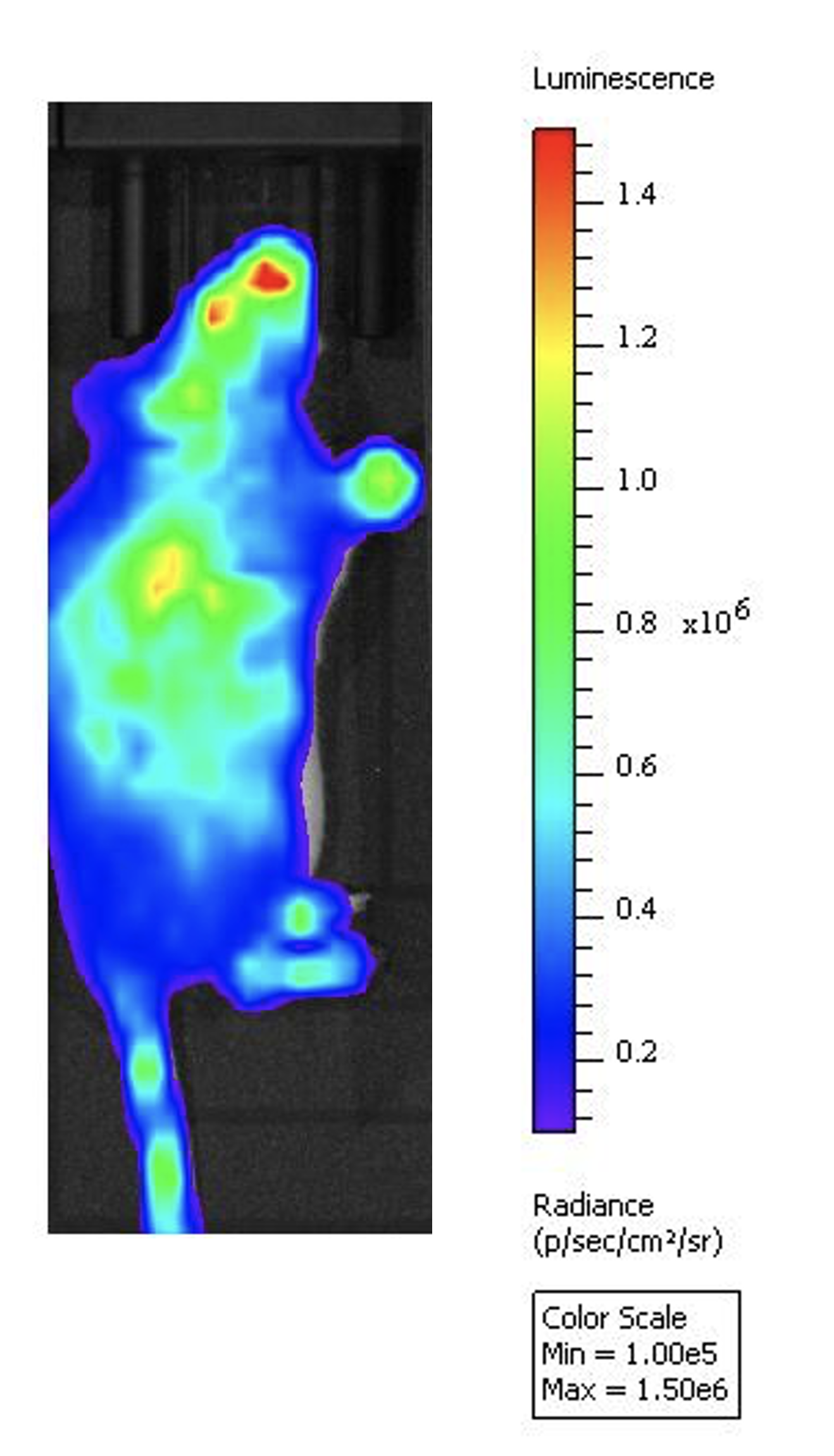

Supplement: S1 Fig — IVIS imagining of NSG mice that had been engrafted with huRBCs was done to confirm parasite luciferase activity. (TIFF) [file pone.0279144.s003.tiff]

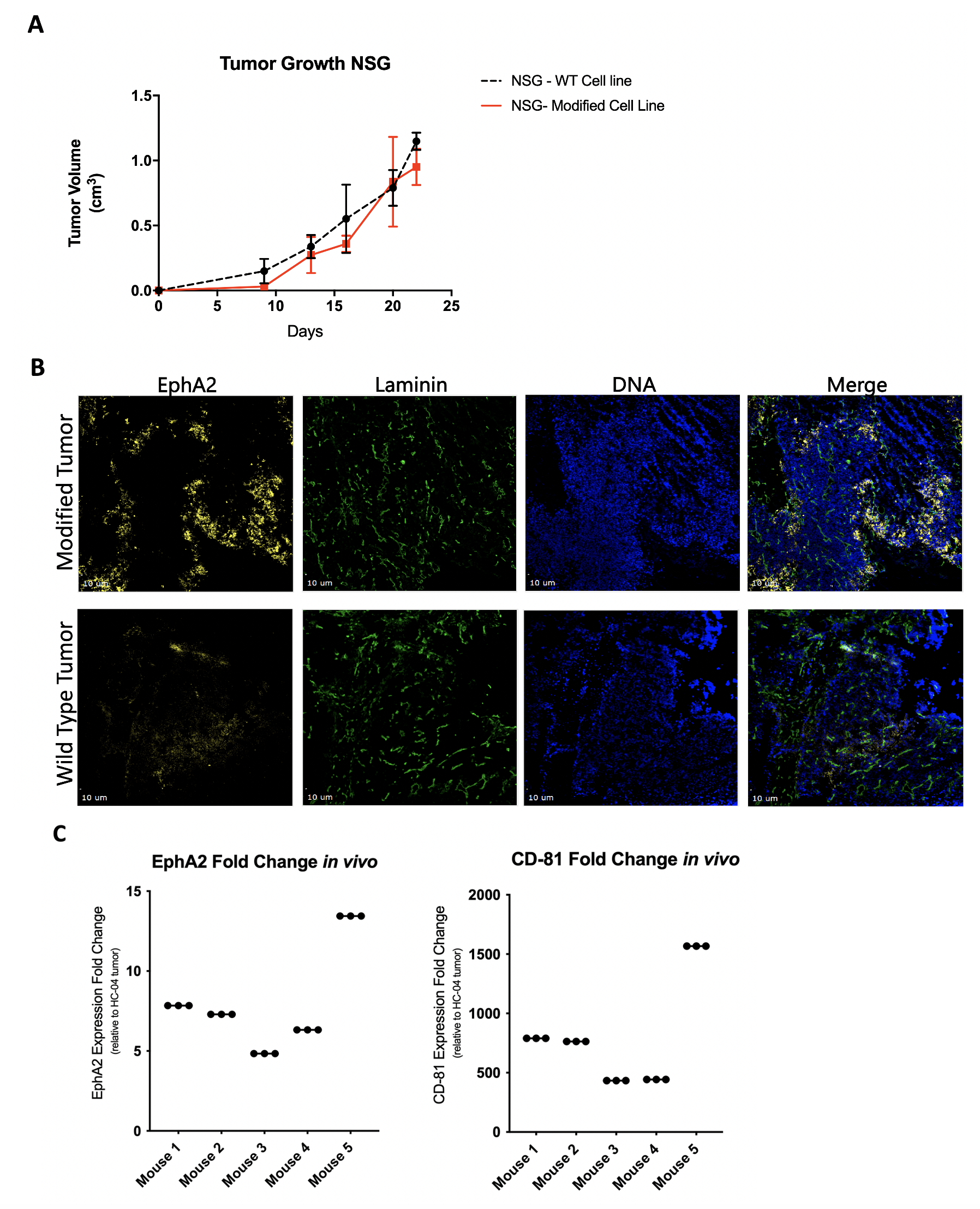

Supplement: S2 Fig — The HC-04 wild type (WT) cell line and HC-04 EphA2High CD81 High modified cell line were compared to assess functionality and receptor expression. (A) Ectopic huLiver growth curve comparison. 5 X106 cells of either HC-04 WT cell line or HC-04 EphA2High CD81 High cell line were injected s.c. on the flank of NSG mice with ectopic huLiver growth monitored daily until day 22 post engraftment. (B) Ectopic huLiver immunofluorescence labeling. Liver sections were assayed by indirect immunofluorescence using the following antibodies: mouse EphA2 APC-conjugated antibody (yellow), polyclonal rabbit anti-laminin Dylight 488 antibody (green) and DNA was visualized using DAPI (blue). (C) EphA2 and CD81 expression levels. EphA2 and CD81 receptor expression levels measured by qRT-PCR, relative to the housekeeping gene GAPDH. (TIFF) [file pone.0279144.s004.tiff]
